# A randomised controlled trial of hearing and vision support in dementia: Protocol for a process evaluation in the SENSE-Cog trial

**DOI:** 10.1186/s13063-020-4135-4

**Published:** 2020-02-24

**Authors:** Iracema Leroi, Christopher J. Armitage, Fidéline Collin, Eric Frison, Mark Hann, Emma Hooper, David Reeves, Zoe Simkin, Lucas Wolski, on behalf of the SENSE-Cog Work Package 3 Investigators, Harvey Abrams, Nathalie Chaghil-Boissière, Anna Pavlina Charalambous, Fidéline Collin, Fofi Constantinidou, Lisa Crosby, Piers Dawes, Eric Frison, Camille Gilbert, Mark Hann, Emma Hooper, Ines Himmelsbach, Evangelia Kontogianni, Brian Lawlor, Iracema Leroi, Sarah Marie, Susana Montecelo, Antonios Politis, Otilia Postea, David Reeves, David Renaud, Zoe Simkin, Monique Termote, Chryssoula Thodi, Lucas Wolski

**Affiliations:** 1 Global Brain Health Institute, School of Medicine, Trinity College Dublin, Trinity College Institute of Neurosciences, Room 0.60, Lloyd Building, Dublin 2, Ireland; 2 Division of Neuroscience and Experimental Psychology, University of Manchester and the Manchester Academic Health Sciences Centre, Manchester, UK; 3 Manchester Centre for Health Psychology, School of Psychological Sciences, University of Manchester, Manchester, UK; 4 Bordeaux Population Health Center, Univ. Bordeaux, INSERM, EUCLID/F-CRIN Clinical Trials Platform, CHU Bordeaux, F-33000 Bordeaux, France; 5 Division of Population Health, Health Services Research & Primary Care, University of Manchester, Manchester, UK; 6 Institute of Applied Research, Development and Continuing Education, Catholic University of Applied Sciences, Freiburg, Germany

**Keywords:** Process evaluation, Complex intervention, Dementia, Sensory impairment, Randomised controlled trial, Hearing impairment, Vision impairment, Mediators, Moderators, Logic model

## Abstract

**Background:**

Optimising hearing and vision function may be important in improving a range of outcomes for people living with dementia (PwD) and their companions. The SENSE-Cog cross-national randomised controlled trial (RCT) is evaluating the effectiveness of a sensory intervention (SI) to improve quality of life for PwD with concurrent hearing and/or vision impairment, in five European countries. To ascertain how or why the intervention will, or will not, achieve its outcomes, we have designed a process evaluation to explore potential discrepancies between expected and observed outcomes. This will also help us to understand how context may influence the outcomes. Here we describe the protocol for this process evaluation, which is embedded within the RCT.

**Methods/design:**

We will use a mixed methods approach with a theoretical framework derived from the UK Medical Research Council’s’ guidance on process evaluations. It will include the following: (1) evaluating how key aspects of the intervention will be *delivered*, which will be important to scale the intervention in real world populations; (2) characterising the *contextual* issues, which may shape the delivery and the impact of the intervention in different countries; and (3) investigating possible *causal mechanisms* through analyses of potential moderators and mediators. To avoid bias, we will analyse the process data before the analysis of the main effectiveness outcomes.

**Discussion:**

This evaluation will provide insight into how the complex SENSE-Cog SI will be tailored, enacted and received across the different European contexts, all of which have unique health and social care economies. The findings will provide insight into the causal mechanisms effecting change, and will determine whether we should implement the intervention, if effective, on a wider scale for PwD and concurrent sensory impairment.

**Trial registration:**

ISRCTN, ISRCTN17056211. Registered on 19 February 2018.

## Background

There is growing evidence that people with dementia (PwD) with ageing-related hearing and vision impairment experience worse outcomes compared to PwD with optimal sensory function. These outcomes include increased disorientation, difficulties self-locating using visual or auditory cues, higher levels of distress leading to agitation and aggression and increased prevalence of hallucinations, delusions and depression [1–3]. Sensory impairment itself can worsen cognitive decline, as well as exacerbate the social isolation that is often associated with dementia [4]. Individuals may withdraw from social activities and hobbies and become marginalised [5–8]. Furthermore, burnout and physical exhaustion in care partners can be amplified by communication barriers [3] and greater dependency of the PwD. Thus, we designed the SENSE-Cog randomised controlled trial (RCT) to answer the research question, ‘Can a home-based, tailored ‘sensory intervention’ (SI) improve quality of life in PwD with comorbid hearing and/or visual impairment?’

The multi-component SI that the SENSE-Cog trial will evaluate comprises assessment, treatment and support of hearing and vision impairment in PwD. We developed the SI iteratively over 24 months. This involved: (1) a scoping review of the literature [9]; (2) an in-depth qualitative exploration of the support care needs of PwD with sensory impairment in three European countries [10]; (3) an international survey (*n* = 653); and (4) an interdisciplinary Expert Reference Group (*n* = 17) [11, 12]. The findings were synthesized into a draft SI that was then field tested in the UK, France and Cyprus [13–15], prior to development of the protocol for the full multi-site RCT [16], the SENSE-Cog trial.

The SENSE-Cog trial is a 36-week parallel-group, observer-blind, multicentre, superiority RCT comparing the individualised SI to usual care in PwD with hearing and/or visual impairment and their companion (the participant ‘dyad’). Briefly, it involves 354 randomized dyads (1:1; 177 per arm) in five European sites: Athens (Greece), Dublin (Ireland), Manchester (UK), Nice (France), and Nicosia (Cyprus). The primary outcome of the trial is quality of life in the PwD, measured at 36 weeks post-baseline using the DEMQOL [17]. The DEMQOL is a 29-item, interviewer-administered, self-report questionnaire with good psychometric properties in persons with mild to moderate dementia. Secondary outcomes include neuropsychiatric symptoms, measures of mental wellbeing, sensory and cognitive functional ability, relationships and health resource utilisation. Companion outcomes and health economic measures are also being assessed. Here we describe the protocol for a process evaluation of the RCT as per the UK Medical Research Council (MRC) recommendations [18].

‘Complex interventions’, such as the SENSE-Cog SI, are defined as those comprising multiple components interacting to produce change [18, 19]. The range of outcomes, and the degree of tailoring or flexibility required for each individual participant, is significant, thus adding to the complexity [12]. While RCTs are the soundest means of inferring causality of an intervention, they cannot ascertain *how* or *why* an intervention may or may not achieve the outcomes [20]. Thus, we have embedded a detailed process evaluation within the RCT to clarify this [18]. This evaluation, together with the outcomes of the RCT, will enable policy makers, funders and practitioners to determine whether the intervention is effective or not, and whether it should be implemented on a wider scale. This is particularly relevant for the cross-national SENSE-Cog trial, which is taking place in five European contexts. It will fill a significant evidence gap in the management of hearing and vision impairment in PwD.

The specific aims of this process evaluation are to: (1) explore the *delivery*, or the process through which the SI will be offered, including barriers and facilitators; (2) evaluate *contextual* issues and clarify factors that may affect the SI delivery, mechanisms and outcomes [21]; and (3) investigate possible *causal mechanisms*, using analyses of potential moderators and mediators.

## Methods/design

The protocol for our process evaluation follows a systematic approach for the design and conduct of the evaluation [18].

### Planning the process evaluation

#### Working with intervention developers and implementers

A process evaluation requires a degree of independence to appraise the intervention team’s delivery of the trial [18]. Thus, we delegated oversight of the evaluation to an expert not involved in the day-to-day conduct of the RCT but on the wider SENSE-Cog team (CA). Regular process evaluation reports will be made at Trial Steering Committee meetings [16] and specific meetings with the study chief investigator (IL), the methodologist (EF) and the process lead (CA).

#### Overlap of the process and outcomes and cost-effectiveness evaluations

Due to the complexity of the study, we have embedded the process evaluation within the daily conduct of the outcomes and cost-effectiveness evaluations. Thus, data for all three purposes are being collected concurrently, and some measures may be used for both process and outcome evaluations. During team training, we emphasized the multi-purposing of data and the need to maintain ‘researcher equipoise’.

#### Description of the intervention

**Fig. 1:**
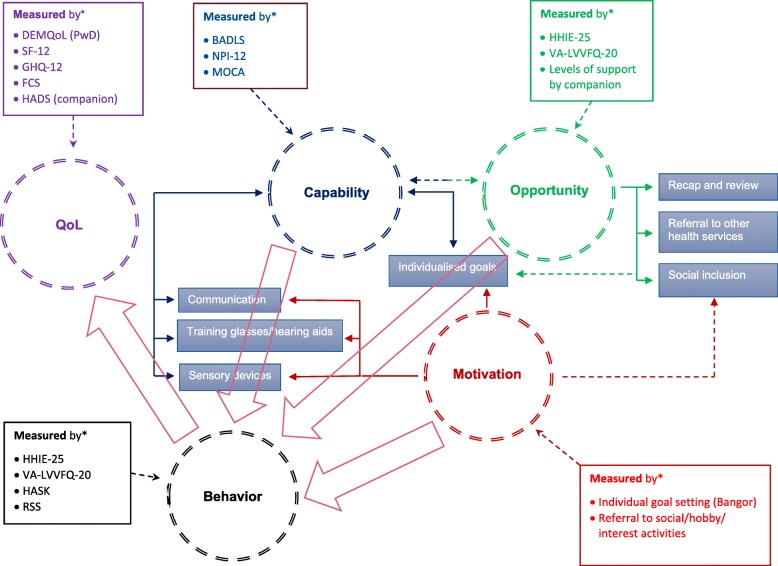
How the elements of the ‘COM-B Behaviour Change’ model and components of the sensory intervention link together. *BADLs* Bristol Activities of Daily Living Scale [22], *Bangor* The Bangor Goal-Setting interview [23], *DEMQoL* Dementia Quality of Life [24], *FCS* Family Caregiving Role Scale [25], *HADS* Hospital Anxiety and Depression Scale [26], *HASK* Hearing Aid Skills and Knowledge test [27], *HHIE-25* Hearing Handicap Inventory for the Elderly [28], *GHQ-12* General Health Questionnaire [29], *MoCA* Montreal Cognitive Assessment [30], *NPI-12* Neuropsychiatric Inventory [31], *RSS* Relationship Satisfaction Scale [32], *SF-12* Short Form Health Survey [33], *VA LV-VFQ-20* Veterans Affairs Low vision Visual Functioning Questionnaire [34]

We have previously detailed the SI components and implementation [12]. In summary, the SI, which is delivered as a ten-session programme by a trained ‘sensory support therapist’ (SST) and audiologists/optometrists, is outlined in Fig. 1. It involves several components, notably: (1) identifying and correcting any vision or hearing impairment; (2) supporting adherence to the hearing and/or vision devices, through advice and training in correct use and care; (3) enhancing communication between the PwD and their companion; (4) demonstrating environmental aids and sensory devices; and (5) accessing relevant support services and social networks. The non-intervention group receives ‘care as usual’ (CAU) access to the services and interventions normally available to PwD and their companions in their respective countries and sites.

#### Causal assumptions about how change will be produced

To understand fully the impact of the intervention on the outcomes, and to generalise the findings in the pan-European context of SENSE-Cog, an exploration of causal mechanisms and unanticipated pathways is needed [19]. We will do this by extracting pre-specified mediating variables and qualitative data (from a sub-sample of 30 participant dyads) and evaluating adverse events and unexpected consequences of the intervention. We will also undertake semi-structured interviews with the SSTs at each of the five study sites.

The principal aim of the intervention is to improve quality of life and functional ability by improving sensory function through devices and behavioural change. Dementia-related quality of life comprises the domains of daily activities (activities of daily living and self-care), physical health and wellbeing, cognitive functioning and social relationships [35]. To influence these domains, we have adopted the COM-B component of the Behaviour Change Wheel [36] as our framework for how the intervention might work. According to this model, behavioural change (‘B’) results from: *capability* (‘C’), the individual’s psychological and physical capacity to engage in the activity concerned; *opportunity* (‘O’), the external factors that support behavioural change; and *motivation* (‘M’), the conscious and sub-conscious processes that direct decision making [36]. In Table 1 and Fig. 1, using a logic model, we outline how each COM-B element aligns with specific components of the SI, and how these might hypothetically lead to improvements in quality of life, our primary outcome.

**Table 1:** Theoretical basis and logic model for how the sensory intervention may impact the person with dementia

Theoretical COM-B domain	Component of the sensory intervention	Behaviour change (B) due to the intervention	Immediate impact of the behaviour change (secondary outcomes of interest)	Intermediate impact of the behaviour change (secondary outcomes of interest)	Primary outcome of interest
*Capability (C)* (the individual’s psychological and physical capacity to engage in the activity concerned)	Correct visual / auditory impairment	• Hear and see better (HHIE-25;VA-LVVFQ-20) • Uptake and adherence improves (HASK) • Greater understanding /insight of impairments • Dyadic interaction improves (RSS) • Enhanced cognitive stimulation	• Enhanced cognitive functioning (MoCA) • Reduced neuropsychiatric symptoms (NPI-12) • Less loneliness in dyad • Enhanced social interactions • Greater independence for person with dementia • Greater companion respite	• Enhanced self-efficacy • Improved communication • Greater functional ability (BADLS) • Reduced apathy (NPI-12) • Relationship satisfaction is higher (RSS) in both person with dementia and companion • Companions health and well-being (SF-12; GHQ-12; FCS; HADS)	• Quality of life in PwD (DEMQoL)
	Training in correct use of hearing aid /glasses with dyad				
	Communication Training with dyad				
	Home-based functional assessment and tailored interventions to address support care needs enabled				
*Opportunity (O)* (external factors which impact on the individual’s ability to ‘live well’ with dementia)	Provision of supplementary sensory devices	• Hear and see better (HHIE-25;VA-LVVFQ-20) • Appropriate levels of support from companion			
	Referral to health and social care services	• Uptake of health and social care services • Uptake of social/hobby/ interest opportunities • Enhanced cognitive stimulation • Achievement of set goals			
	Referral to social/hobby/interest activities				
*Motivation (M)* (in dementia, apathy and loss of motivation is prevalent and may make behavioural change difficult)	Individualised goal setting				
	Referral to social/hobby/ interest activities	• Enhanced uptake and adherence of sensory aids and suggested social opportunities • Uptake of meaningful and enjoyable social/hobby/interest activities			

*BADLs* Bristol Activities of Daily Living Scale [22], *DEMQoL* Dementia Quality of Life [24], *FCS* Family Caregiving Role Scale [25], *HADS* Hospital Anxiety and Depression Scale [26], *HASK* Hearing Aid Skills and Knowledge Test [27], *HHIE-25* Hearing Handicap Inventory for the Elderly [28], *GHQ-12* General Health Questionnaire [29], *MoCA* Montreal Cognitive Assessment [30], *NPI-12* Neuropsychiatric Inventory [31], *RSS* Relationship Satisfaction Scale [32], *SF-12* Short Form Health Survey [33], *VA LV-VFQ-20* Veterans Affairs Low vision Visual Functioning Questionnaire [34]

Briefly, for *capability*, the sensory aids (i.e. hearing aids, glasses and sensory environment modification in the home) and SST adherence support will improve hearing and vision (*physical capability*), which will enhance sensory-cognitive function and overall functional ability and reduce neuropsychiatric symptoms [37–41]. The SST will train communication skills and improve knowledge of dementia and sensory impairment (*psychological capability*). This will increase *opportunity* by decreasing dependency on companions, enhancing social interactions and reducing loneliness. Adherence support for PwD with sensory devices will enhance hearing [42], vision [43] or both [15]. Furthermore, social opportunities will be enhanced through signposting outside the home, thus addressing social isolation, improving social relationships and providing respite for companions. Regarding *motivation*, higher sensory-cognitive function and improved neuropsychiatric symptoms will improve self-efficacy, self-esteem and mental wellbeing [44, 45]. However, motivation may be reduced in dementia, particularly if apathy is present. Thus, the SI will address this through goal setting. We will measure apathy to take account of motivation as a potential moderator, or even mediator, of the intervention’s impact. Greater independence and communication ability in the PwD will reduce companions’ burden and stress, which will also impact positively the overall wellbeing. Attitudes and knowledge training will support change maintenance and relationship quality [46].

#### Identification of key uncertainties and developing a framework for the process evaluation

In Table 2, we identified key uncertainties to address for each SI. We will use a mixed method approach to capture the data for the evaluation. This will involve a variety of instruments, as outlined in Table 3.

**Table 2:** Identifying key uncertainties to address in the process evaluation for each component of the sensory support intervention

Sensory support intervention component	Key uncertainty to address
Correct visual/auditory impairment	Is vision/hearing impairment actually corrected?
Training in correct use of hearing aid/glasses	How well are the devices used by the PwD?
Communication training	Is the companion utilising the techniques?
Home-based functional assessment	What are the types and extent of functional impairment identified by the sensory support therapist?
Referral to health and social care services	What types of services are available at each site? How many referrals were made and actioned?
Provision of supplementary sensory devices in the home environment	How many of these were supplied and how often and effectively were they used?
Referral to social/hobby/interest activities	How many referrals were made and actioned?
Individualised goal setting	What is the number and types of goals set with the participant? How many goals were achieved? What is the level of apathy present (ascertained only after final assessment data collected?

**Table 3:** Measurement instruments used to collect quantitative and qualitative data to inform the process evaluation

Measurement tool		Data captured by whom? About whom/what?	Timing of data capture	Process evaluation (Capability ´C´, Opportunity ´O´, Motivation ´M´) and measurement instruments (details)
1a)	Sociodemographic information	Researcher about dyad	Screening	To assess characteristics of companion and participant (covariates of age, gender, living conditions influencing outcomes)
1b)	Medical history	Researcher about dyad	Screening	Dementia sub-type and medication may influence uptake and effectiveness of intervention (eliciting the type of memory impairment and the current medication)
1c)	HearCheck PEEK acuity, visual field confrontation test	Researcher about PwD	Screening	To establish the baseline for ‘C’ and ‘O’ for intervention/optimisation of function (screening for severity of visual and hearing loss of participant)
1d)	MoCA	Researcher about PwD	Screening	To ascertain impact of sensory optimisation on cognitive ability as intermediate step (‘C’ and ‘M’) leading to improved QoL (assessing cognitive level of participant scale (score ≥ 10); also decides whether the participant is appropriate candidate for the study)
1e)	DEMQoL	Researcher about PwD	Baseline	The primary outcome of the behavioural change process, resulting from intermediate impacts (assessing quality of life of PwD by addressing all parts of the process evaluation: ´C´, ´O´, ´M´)
1f)	BADLs	Companion about PwD	Baseline	To ascertain impact of sensory optimisation on functional ability as intermediate step (‘C’ and ‘M’) leading to improved QoL (assessing functional ability of PwD)
1g)	VA LV-VFQ-20	Researcher about PwD and companion	Baseline	Improvement in sensory input affects ‘C’, ‘O’ and ‘M’, all impacting on intermediate outcomes leading to overall improvement in QoL (assessing for vision- and hearing-related functional ability)
1 h)	HHIE-25	Researcher about PwD and companion	Baseline	
1i)	NPI-12	Researcher and companion about PwD	Baseline	To ascertain impact of sensory optimisation on neuropsychiatric function as intermediate step (‘C’, ‘O’ and ‘M’) leading to improved QoL (assessing behavioural and psychological symptoms like anxiety, agitation etc.)
1j)	RSS	Researcher about PwD (companion completes on its own)	Baseline, week 18, week 36	Improved dyadic relationship optimises ‘O’, thus leading to improved QoL (capturing current level of relationship satisfaction between the PwD and study companion, reduce feelings of loneliness)
1 k)	EQ-5D-5 L	Researcher about PwD and companion	Baseline	To measure uptake of health services, enhancing ‘O’ (assessing health resource utilisation and capturing the amount and type of support that is in place)
1 l)	RUD-Lite	Researcher and companion about PwD	Baseline, week 18, week 36	
1 m)	PwD full visual and/or hearing assessment	By optometrist and/or audiologist about PwD	Following randomisation	To establish the baseline for ‘C’ and ‘O’ (assessing the degree of the PwD’s visual and/or hearing loss)
2)	Delivered devices record	By researcher/therapist about PwD	Following receipt of glasses and/or hearing aids	To ascertain the ‘dose’ of the intervention (capturing the number and type of corrective sensory devices prescribed by the optometrist and/or audiologist)
3a)	PwD diary (acceptability and tolerability of therapy)	By PwD about their own experiences	Throughout sensory intervention	To ascertain the utility of the ‘correction’ component of the intervention from the PwD’s perspective (capturing the PwD’s ratings on acceptability and tolerability of the SI and acceptability across five indices (helpfulness, fatigue, effort, understanding and motivation) as well as the PwD’s view about their corrective sensory devices—also informing ´C´)
3b)	PwD diary (acceptance of devices)	By PwD about their own experiences	Throughout sensory intervention	
3c)	Companion diary (acceptability of therapy)	By companion about PwD’s experiences	Throughout sensory intervention	To ascertain the utility of the ‘correction’ component of the intervention from the companion’s perspective (capturing companions’ ratings on the PwD’s response to the session across five indices of therapy acceptability (interest, autonomy, motivation, emotional responses and mastery) recorded after each session)
3d)	Companion diary (adaptation to devices)	By companion about PwD’s experiences	Throughout sensory intervention	To ascertain uptake/adherence with the corrective devices; to assess level of inter-site variability (capturing companions’ ratings on the PwD’s adaptation to and management and use of the corrective sensory devices)
3e)	Companion diary (companion’s confidence)	By companion about their own experiences	Throughout sensory intervention	To capture the companion training component of the intervention; to assess level of inter-site variability (capturing companion’s confidence in supporting the PwD’s use of their corrective sensory devices)
4a)	SST logbook (visit record)	By therapist about SI visits	Throughout sensory intervention	To ascertain dose of the intervention; to assess level of inter-site variability by: - Measuring the dose of the SI (number of sessions, duration of each session and frequency) - Quantifying the PwD’s capability, opportunity and motivation to use their glasses/hearing aids -Capture adherence to the protocol, i.e. which aspects of the SI were delivered by the SST
4b)	SST logbook (response to corrective devices)	By therapist about PwD	Throughout sensory intervention	
4c)	SST logbook (delivered components record)	By therapist about SI visits	Throughout sensory intervention	
4d)	Bangor Goal Setting Inventory	By therapist about PwD	End of sensory intervention	To measure little changes within ´M´ (capturing the number and type of goals set by the PwD, and their progress towards these during the SI—address support care needs)
4e)	Hearing aid skills and knowledge test and/or glasses/vision skills and knowledge test	By therapist about PwD	Following receipt of hearing aids/glasses and at end of sensory intervention	To enhance self-efficacy both in PwD and companion: ´C´ and ´O´ (capturing the change in the PwD and companion’s skills and knowledge in using their corrective sensory devices through receiving the SI)
4f)	GHABP	By audiologist and therapist about PwD	At audiology assessment and at end of sensory intervention	To ascertain improved dyadic communication outcomes: ´C´(capturing self-reported gains from hearing aid use)
5)	Dyadic experience of SI (interview)	By researcher about PwD and companion	Intervention end	To ascertain how the interviews with the dyads were experienced (reflecting upon the positive and negative experiences of intervention for the dyads, perceived benefits/short-comings, suggestions for improvement)
6)	Battery of outcome measures	By researcher about PwD	Baseline, week 18, week 36	The secondary outcome of the support intervention focusing on behavioural changes (assessing outcomes for participants and companions)
7a)	SST knowledge and skills checklist	By therapy supervisor about each therapist	Prior to and post-SST training	To assess level of inter-site variability as to the SST by: - A 13-item self-rated questionnaire to identify existing knowledge and skills necessary for the SST role and training needs pre and post-SST training) - Verifying that the SST has received training in the delivery of the SI components) - Verify that the SST is competent to undertake the role - Capturing the number, duration and essence of supervision provided by the senior SST - Assessing positive and negative aspects of intervention, emerging adaptations, suggestions for improvement
7b)	SST training log	By therapy supervisor about each therapist	Prior to trial commencement	
7c)	SST competency checklist	By therapy supervisor about each therapist	Prior to trial commencement	
7d)	SST supervision log and reflective diary	By each therapist and therapy supervisor about themselves	Throughout trial intervention	

*MoCA* Montreal Cognitive Assessment [30], *DEMQoL* Dementia Quality of Life [24], *BADLs* Bristol Activities of Daily Living Scale [22], *VA LV-VFQ-20* Veterans Affairs Low vision Visual Functioning Questionnaire-20 items [34], *HHIE-25* Hearing Handicap Inventory for the Elderly-25 items [28], *NPI-12* Neuropsychiatric Inventory-12 items [31], *RSS* Relationship Satisfaction Scale [32], *EQ-5D-5 L* 5-level EuroQol 5-dimension [47], *RUD-Lite* Resource Utilization in Dementia-Lite [48], *GHABP* Glasgow Hearing Aid Benefit Profile [49]

#### Exploration of delivery

To ascertain whether the SI is delivered (i.e. ‘how’) and enacted (i.e. ‘what’) as intended [18], we will examine the fidelity and dose (i.e. duration, number and frequency of SI visits) of the delivered intervention. Due to potential burden on participants, we have chosen *not* to include an external evaluation of fidelity (i.e. independent observer during sessions). Instead, we will rely on the proxy measure of ‘fidelity’ as determined by thoroughness of SST training and supervision, use of therapist manual and SST logbook recordings of sessions. We will document the nature of the support offered by the intervention, including the type of corrective devices, the environmental changes to support sensory function, the number and types of referral or signposts to extra-trial services. These data will be captured through participant diaries (the PwD and their companion) and the SST logbook.

Specifically, the *PwD diaries* will contain Likert style ratings [50] of acceptability and tolerability of SI visits, including measures of helpfulness, effort, fatigue, understanding and motivation; and how acceptable the corrective sensory devices are. The *companions´ diaries* will capture data relating to how the PwD engages with the visit, how the PwD is adapting to their sensory aids, and how confident the companion feels in supporting the PwD in using the aids. The *SST logbooks* will contain details of each visit, the components of the SI delivered, participant response to the intervention and skill in managing their aids. Additionally, the SST logbooks will detail how the SI is specifically tailored to the dyad.

We will assess *reach* through the representativeness of the sites, the recruitment process (refusal rate, attrition rate) and the representativeness of the study population according to the target population [51].

#### Evaluation of contextual issues

The SENSE-Cog RCT will take place in several different countries and involve three languages (English, French and Greek). Thus, contextual issues, which are external to the intervention itself, need to be carefully considered. These include differences in language, culture, access to services and the health and social care economy. Context may influence the SST’s ability to foster change in the participant dyad’s circumstances. For example, for social isolation, the SST may recommend attendance at a local lunch club; however, if transportation is not suitable for individuals with sensory and cognitive impairment, the opportunity to take up the offer will be hampered. Likewise, communication training with companions may be differently received in diverse cultural and linguistic contexts. Thus, the same intervention may have divergent outcomes according to the setting in which it is delivered [18]. The dyadic relationship (between the PwD and their companion) should also be considered because the level of support and quality of relationship may vary among dyads. To capture contextual data, we will collect information from the demographic and outcome measures, the participant dyad diaries, the SST logbook and in-depth qualitative interviews of a sub-sample of participant dyads (*n* = 30 dyads across the sites), as detailed in the SENSE-Cog trial protocol [16].

#### Sampling and timing of data collection

We will collect characteristics of each participant dyad, including gender, age and support structure at the screening visit, and at baseline, week 18 and week 36 (Tables 3 and 4). Following each SI visit (for the active arm), participant diaries and SST logbooks will be completed. The sub-sample qualitative interviews will take place within 2 weeks following the SI (details described in [16]). Training logs for the SSTs were collected prior to study start. SST supervision logs and fidelity checks of the SST logbooks are being collected throughout the trial. SST interviews will be held within 2 weeks after the last intervention visit of the last randomised dyad in each site. Briefly, these interviews will explore the experience of having received the intervention, from the perspective of each member of the dyad. The sample size for this sub-sample was selected to achieve theoretical and data saturation. The interviews from all sites will be analysed by using conventional qualitative content analysis [52] and a grounded theory approach [53].

**Table 4:**
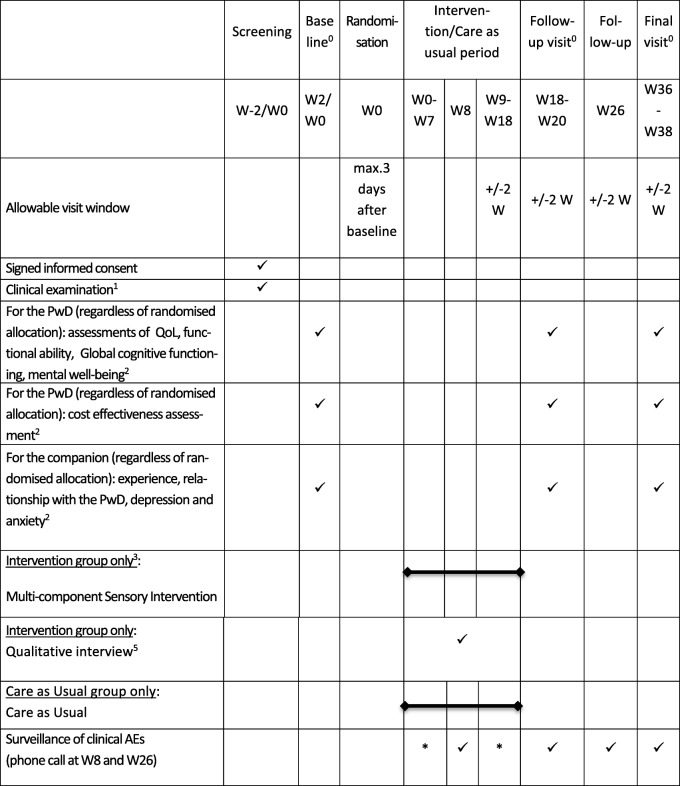
Participant assessment and follow-up visit schedule for the SENSE-Cog Trial

^0^Baseline, follow-up and end visits may be split into two visits, occurring within a maximum of a 2-week window according to the PwD’s needs

^1^Clinical examination including assessment of vision impairment (using the PEEK tool), hearing impairment (using the HearCheck device), level of cognitive impairment and other eligibility criteria and medical diagnostic of depression

^2^ Baseline (except for the MoCA scale, which will be performed at screening), W18 and W36 (around 2 to 2.5 h)

^3^The number of sessions may vary from participant to participant but the maximum number of visits will be ten at an average rate of one per week. The order and duration of each component may vary according to the participants’ needs, as determined in collaboration with the researcher, the PwD and the companion

The remaining weeks until week 18 will allow the SST to revisit and recap previous components, based on the participant’s individual needs and goal attainment

^4^In case of dual impairment, the hearing assessment will be done first, followed by the vision assessment, if possible

^5^A qualitative interview will be consecutively proposed to each dyad:

- Who experienced completed SI (all intervention visits scheduled by the SST, in addition to the full hearing and vision assessments)

- at the therapist discretion based on their clinical impression of the person be able to provide meaningful feedback

- and who are willing to

- until we reach participation of 60 people (30 interviews), six dyads per site: four dyads with single impairment (two hearing only and two vision only if possible) and two dyads with dual impairment

This qualitative interview will take place within 2 weeks of SI completion. One additional interview, if possible, will be undertaken with a dyad which did not complete the full SI

*W* week

### Analysis

To avoid biased interpretation, as recommended by the MRC’s guidance, we will analyse and explore process data arising from the qualitative interviews and contextual factors before the unblinded trial outcomes are known [18]. We will use the process data to generate specific hypotheses (pre-trial explanation before trial outcomes are revealed) regarding factors that moderate and/or mediate the effect of the SI on outcomes, notably quality of life. This will minimise the risk of ‘fishing’ for relationships and falsely significant findings due to multiple testing.

Moderator analyses will be undertaken, with appropriate caution, to investigate any influence of the baseline characteristics of the dyads (e.g. age, gender, type of sensory impairment, level of cognitive impairment, type of companion) and country/site effect on the strength and/or direction of the relationship between the SI and the outcomes. We will undertake mediation analyses to assess the degree to which the impact of the SI on the stated outcomes is a direct effect, or is indirect via the hypothesised mediating factors which will be modelled as latent variables in a structural equation modelling framework.

We will conduct the moderator/mediator analysis only after the final RCT analysis has been completed and the dataset has been un-blinded. We will do this regardless of whether the SI has a significant direct impact on the primary outcome.

We will apply a regression framework, using newer methods and statistical models [54, 55] that improve on traditional approaches (e.g. [56]). These models can become complex, particularly when controlling for multiple covariates. Thus, depending upon the number and complexity of the hypotheses to be tested, we will assess whether it will be better to analyse each factor separately, or to combine sets of moderators and/or mediators into a ‘conditional process analysis’ [54]. We will conduct separate analyses for those variables available in both trial arms, and those available in the intervention group only (e.g. related to the SI). The latter analyses will help to identify process measures that are part of the SI and may moderate its efficacy (for e.g., number of SST visits, SST experience and fidelity), using appropriate techniques [57].

## Discussion

The process evaluation of the SENSE-Cog RCT will appraise several important aspects of the delivery of the intervention, the context of delivery and the hypothesised causative mechanisms. These issues are key to interpreting the effectiveness outcomes of the trial and, if outcomes are positive, to assist in understanding implications for scale-up in clinical settings. The SI will have multiple interacting components: assessing and correcting hearing and vision impairment (hearing aids and/or glasses lenses), training in the use of the devices, enhancing communication within the dyad, optimising the home sensory environment and supporting engagement in health and wellbeing opportunities in the community, including social integration and external support services.

A key challenge in delivering the SENSE-Cog SI is to maintain standardisation and rigour when implementing such a complex intervention across five different European sites. The SENSE-Cog SI is ambitious in its vision in addressing three co-morbidities simultaneously—cognitive impairment, hearing loss and vision loss—and to assess the impact of a psychosocial intervention on managing these impairments. We will aim to capture the cultural, social and economic nuances of the respective European study sites whilst deriving results that can be applied in a pan-European context. To ensure that we capture the cultural differences from the qualitative interviews, we will keep translation to a minimum, as recommended by Haak et al. [58].

The theoretical model with causal relationships will be informed by the mediation analyses. The elicited data will also enable us to ‘test’ the theoretical model of the COM-B by addressing the key uncertainties listed in Table 1. This will be the first opportunity to evaluate empirically the COM-B model in a RCT setting, as demonstrated in Fig. 1.

### Strengths and limitations of the study

A strength of our study protocol includes the use of a mixed method approach, including both qualitative and quantitative measures, to carefully explore the ‘how and why’ of the intervention. Another strength of our approach includes the sound theoretical framework on which the intervention was developed, and the iterative manner in which it was modified and field-tested [12, 15] before arriving at the final version of the intervention, ready for full scale effectiveness testing. A limitation (although also a potential strength) is the significant degree of variability in the study sites due to the different EU contexts in which the programme takes place, as well as the variability of the intervention offered to each participant dyad, resulting from the tailored approach.

### Reporting and dissemination

We will report the results of the process evaluation described here using a combination of reporting guidance, including CONSORT [59] and COREQ (for the qualitative outcomes) [60] as well as statistical methods for mediators and moderators [61]. We will submit the findings to an open-access journal, as per the requirements of the funder. As recommended by others [51], we have described our protocol in advance to foster transparency in reporting and to help the development and evaluation of complex psychosocial interventions for PwD, an emerging area of health services research. Finally, we will link our outputs related to the SENSE-Cog trial through the SENSE-Cog programme website (www.sense-cog.eu).

### Ethics approval and consent to participate

In Manchester, the study received final approval (version 3.0) by the NW Haydock ethics committee on 22 January 2018 and obtained sponsor approval on 8 March 2019. In Nicosia, the study received favourable opinion on 27 September 2016 from the Cyprus National Bioethics Committee. In Athens, the Local Ethics Committee of Health Sciences and Scientific Committee of the Eginition Hospital of the National and Kapodistrian University of Athens ethics committee granted a favourable opinion on 24 January 2018. In Dublin, the Saint James Hospital/AMNCH Research Ethics Committee gave approval on the 25 October 2018. In Nice, the “Comité de Protection des personnes Sud Est I” gave a favourable opinion on 12 July 2018. Written consent is collected from the participants eligible for the study, using procedures in accordance with the national guidance regarding informed consent and clinical research (for individuals with or without capacity to consent) in each of the participating countries (detailed in Regan et al. [16]). All researchers have been fully trained in Good Clinical Practice (GCP) and mental capacity assessment skills and follow national guidance in their respective countries, such as the Mental Capacity Act (2005) in the UK. If a person lacks capacity, a nominated consultee will be asked to deem whether it is in the PwD’s best interests to participate.

### Trial status

This process evaluation is based on the SENSE-Cog RCT protocol version 4.0 of 16 November 2018. The overall SENSE-Cog research programme started in January 2016 and the SENSE-Cog RCT (Work Package 3.2) started recruitment in summer 2018. Recruitment is expected to end in December 2020. The first qualitative interviews with participants took place in November 2018. The process evaluation will start in following the last W36 assessment of the first randomized participant dyad.

## Supplementary information

**Additional file 1.** Good Reporting of A Mixed Methods Study (GRAMMS).

## Data Availability

Data generated or analysed during this study will be included in the article reporting the results that will be shared through scientific articles and international conferences.

## Abbreviations

AE: Adverse events
COM-B: Capability, opportunity, motivation-behavioural change wheel
MRC: Medical research council
PwD: People with dementia
RCT: Randomized controlled trial
SI: Sensory intervention
SST: Sensory support therapist
