# Supplementary material for: A randomised controlled trial of hearing and vision support in dementia: Protocol for a process evaluation in the SENSE-Cog trial
Source: Trials. 2020 Feb 24;21:223. doi: 10.1186/s13063-020-4135-4 (PMC7041097; doi:10.1186/s13063-020-4135-4)
Supplement: Supplementary file 1 — Additional file 1. Good Reporting of A Mixed Methods Study (GRAMMS). [file 13063_2020_4135_MOESM1_ESM.docx]

**Good Reporting of A Mixed Methods Study (GRAMMS)**

| **Guideline** | **Section: page** |
| --- | --- |
| Describe the justification for using a mixed methods approach to the research question | Strength and Limitations: p.9 |
| Describe the design in terms of the purpose, priority and sequence of methods | p. 4-8 |
| Describe each method in terms of sampling, data collection and analysis | p. 8 |
| Describe where integration has occurred, how it has occurred and who has participated in it | p. 4 |
| Describe any limitation of one method associated with the present of the other method | N.A. |
| Describe any insights gained from mixing or integrating methods | p. 6? |

O'Cathain A, Murphy E, Nicholl J. The quality of mixed methods studies in health services research. J Health Serv Res Policy. 2008;13(2):92-98 doi: 10.1258/jhsrp.2007.007074.
